# Altered habenular and whole brain functional connectivity in early Parkinson’s disease using 7 T MRI

**DOI:** 10.1038/s41531-025-00973-6

**Published:** 2025-10-01

**Authors:** Bedia Samanci, Ulas Ay, Mark L. Kuijf, Sonny Tan, Yasin Temel

**Affiliations:** 1 School for Mental Health and Neurosciences, Maastricht University, Maastricht, The Netherlands; 2 Behavioral Neurology and Movement Disorders Unit, Department of Neurology, Istanbul Faculty of Medicine, Istanbul University, Istanbul, Turkey; 3 Neuroimaging Unit, Hulusi Behçet Life Sciences Research Laboratory, Istanbul University, Istanbul, Turkey; 4 Department of Physiology, Istanbul Faculty of Medicine, Istanbul University, Istanbul, Turkey; 5 Department of Neurology, Maastricht University Medical Centre, Maastricht, The Netherlands; 6 Department of Neurosurgery, Antwerp University Hospital, University of Antwerp, Edegem, Belgium; 7 Translational Neurosciences, Faculty of Medicine and Health Sciences, University of Antwerp, Antwerp, Belgium; 8 Department of Neurosurgery, Maastricht University Medical Centre, Maastricht, The Netherlands; 9 Atlas University, Istanbul, Turkey

**Keywords:** Parkinson's disease, Functional magnetic resonance imaging

## Abstract

Parkinson’s disease (PD) is traditionally linked to basal ganglia dysfunction, yet evidence highlights broader network disruptions. Habenula, involved in regulating mood, reward, and motor functions, remains underexplored in PD. This study investigated whole-brain and habenular functional connectivity and their clinical correlates in early-stage PD using 7 T MRI. Functional connectivity was analyzed in 104 early-stage PD and 45 healthy controls. Whole-brain analysis revealed increased connectivity in two clusters in PD: the first involved paracentral lobule, middle frontal gyrus, orbital middle frontal gyrus, precentral gyrus, angular gyrus, middle cingulate gyrus, supplementary motor area; the second included middle cingulate gyrus and cerebellum Crus I. Left habenula showed increased connectivity with right middle temporal and angular gyri (p-FDR = 0.011). Levodopa equivalent daily dose positively correlated with connectivity between postcentral gyrus and cerebellum (p-FDR = 0.020). These findings highlight early motor and cognitive network disruptions and suggest these regions may serve as potential markers of PD-related neurobiological changes.

## Introduction

Parkinson’s disease (PD) is traditionally characterized as a movement disorder resulting from significant loss of dopaminergic neurons in the nigrostriatal pathway. However, it is well known that PD also encompasses a wide range of non-motor symptoms, including cognitive impairment and mood disorders, which may involve alterations in multiple brain regions implicated in cognitive and emotional processing^1^. This makes PD clinically and etiologically diverse, and its complex neurobiological foundations are not yet fully understood.

The pathophysiology of PD extends beyond the dopaminergic system, involving multiple neural pathways and brain regions. Studies using resting-state functional magnetic resonance imaging (rs-fMRI) have identified alterations in several large-scale brain networks implicated in both motor and non-motor symptoms of PD, including the default mode network (DMN), sensorimotor network (SMN), salience network (SN), and fronto-parietal network (FPN)^2^. Specifically, disruptions within the SMN have been associated with motor symptom severity, while DMN alterations are linked to cognitive decline, and SN abnormalities correlate with mood disturbances in PD. Among these networks, intrinsic dysfunction within the dorsolateral prefrontal cortex (PFC) has been observed in depressed PD (dPD) patients, suggesting its involvement in mood and executive function impairments^3,4^. Similarly, apathy in PD has been associated with disrupted frontostriatal connectivity, which affects motivation and emotional processing^5,6^. A meta-analysis further revealed that decreased functional connectivity within the DMN consistently differentiates cognitively impaired and unimpaired PD patients^7^. In early-stage, cognitively unimpaired PD patients, decreased medial temporal and inferior parietal connectivity within the DMN correlated with cognitive performance, suggesting that posterior brain disconnection may precede cognitive impairment in PD^8^. Recent studies suggest that these network alterations are not merely a consequence of dopaminergic degeneration but may represent compensatory mechanisms or early pathophysiological changes. For example, structural and functional alterations in the cerebellum have been observed in early-stage PD, indicating that cerebellar changes may contribute to the disease process from its earliest phases^9^. Additionally, alterations in cortical motor areas have been linked to cerebellar hyperconnectivity in early to moderately advanced PD, potentially reflecting compensatory mechanisms for functional recovery^10^. It should also be noted that dopaminergic therapy, particularly levodopa, plays a crucial role in symptom management but has been shown to modulate functional connectivity across both motor and non-motor networks^11,12^.

Given the widespread network alterations observed in PD, it is crucial to explore how subcortical structures, such as the habenula (Hb), contribute to both motor and non-motor dysfunctions, particularly in the early stages of the disease. The Hb is a small pair of nuclei located adjacent to the dorsomedial thalamus, with extensive connections to cortical (dorsal anterior cingulate cortex, medial PFC, left inferior frontal sulcus, middle and posterior anterior cingulate, retrosplenial cortex, primary visual and auditory cortices, and posterior insula) and subcortical (thalamus, putamen, caudate, bed nucleus of the stria terminalis, nucleus basalis of Meynert, posterior hippocampus, septal nuclei, periaqueductal gray, dorsal raphe nuclei, ventral tegmental area and parahippocampal gyrus)^13,14^. It plays a key role in reward processing, learning, motivation, pain perception, aversion, and motor functions^15–18^. Recently, it has gained increasing attention for its role in mood and reward regulation, primarily through its influence on dopaminergic and serotonergic pathways. Given its widespread connectivity, dysfunction of the Hb has been implicated in mood disorders in both PD and non-PD populations; however, functional connectivity changes in the Hb remain underexplored in early-stage PD patients without clinically diagnosed depression^19–23^.

In this study, we aimed to investigate (a) whole brain functional connectivity, (b) the functional connectivity between the Hb and the whole brain, and (c) the relationship between connectivity changes and clinical features in early-stage PD patients compared to healthy controls (HCs) using ultra-high field 7 T fMRI data from the longitudinal observational TRACK-PD study designed to investigate functional and structural brain changes in early-stage PD^24^. 7 T MRI offers superior spatial resolution and signal-to-noise ratio compared to conventional field strengths. These advantages enable precise mapping of small structures like the Hb and allow differentiation between its medial and lateral nuclei, enhancing the detection of subtle functional connectivity changes in early PD^25^. We hypothesize that there will be significant differences in whole brain and Hb connectivity between PD patients and HCs, and that these differences will correlate significantly with clinical features, reflecting early neurobiological changes in PD.

## Results

### Clinical and demographical results

The PD and HC groups were matched for age, sex, handedness, and educational status. Among the PD patients, 32 (30.8%) were categorized as H&Y stage 1, 68 (65.4%) as stage 2, and 4 (3.8%) as stage 3.

A Kruskal-Wallis H-test revealed no significant differences in BDI total scores (*p* = 0.092), MoCA total scores (*p* = 0.203), or LEDD (0.682) across different H&Y stage 1 (*n* = 32), stage 2 (*n* = 68), and stage 3 (*n* = 4) groups. However, within the PD group, BDI total scores showed a significant positive correlation with both LEDD (*r* = 0.274, *p* = 0.005) and disease duration (r = 0.222, *p* = 0.024). Additionally, a significant negative correlation was found between BDI total scores and MoCA total scores (*r* = −0.242, *p* = 0.013). Conversely, no correlation was identified between BDI total scores and MDS-UPDRS Part III scores.

The mean BDI total score was significantly higher in the PD group compared to HCs (7.3 ± 5.07 vs. 3.5 ± 3.60; *p* < 0.001) (Table 1). Among PD patients, 7 (6.7%) were diagnosed with significant depression, 34 (32.7%) had SubD, and 63 (60.6%) exhibited no depression based on BDI scores. In the HC group, none were classified with significant depression, 5 participants (11.1%) fell into the SubD category, and 40 (88.9%) showed no signs of depression. Antidepressant or anxiolytic treatments were used by 5 PD patients (2 selective serotonin reuptake inhibitors (SSRIs), 1 amitriptyline, 1 serotonin-noradrenaline reuptake inhibitor, and 1 oxazepam) and 2 HCs (both on SSRIs).

**Table 1:** Clinical and demographic data of the participants

	PD group (n = 104)	HC group (n = 45)	p
**Sex (M/F, n)**	73/31	31/14	0.874
**Handedness (R/L, n)**	89/15	42/3	0.182
**Age (years)**	62.4 ± 8.45	60.7 ± 8.07	0.240
**Disease Duration (month)**	19.7 ± 9.50	-	N/A
**LEDD**	402.2 ± 230.5	-	N/A
**MDS-UPDRS Part III Score**	19.1 ± 7.48	-	N/A
**MoCA Total Score**	27.9 ± 1.63	27.7 ± 1.56	0.634
**BDI Total Score**	7.3 ± 5.07	3.5 ± 3.60	<0.001

PD: Parkinson’s disease, HC: Healthy control, M: Male, F: Female, R: Right, L: Left, LEDD: Levodopa equivalent daily dose, MDS-UPDRS: Movement Disorders Society-Unified Parkinson’s Disease Rating Scale, MOCA: Montreal Cognitive Assessment, BDI: Beck Depression Inventory.

The clinical and demographic data of the participants are presented in Table 1.

### Comparison of HC and PD groups in whole brain functional connectivity

**Fig. 1:**
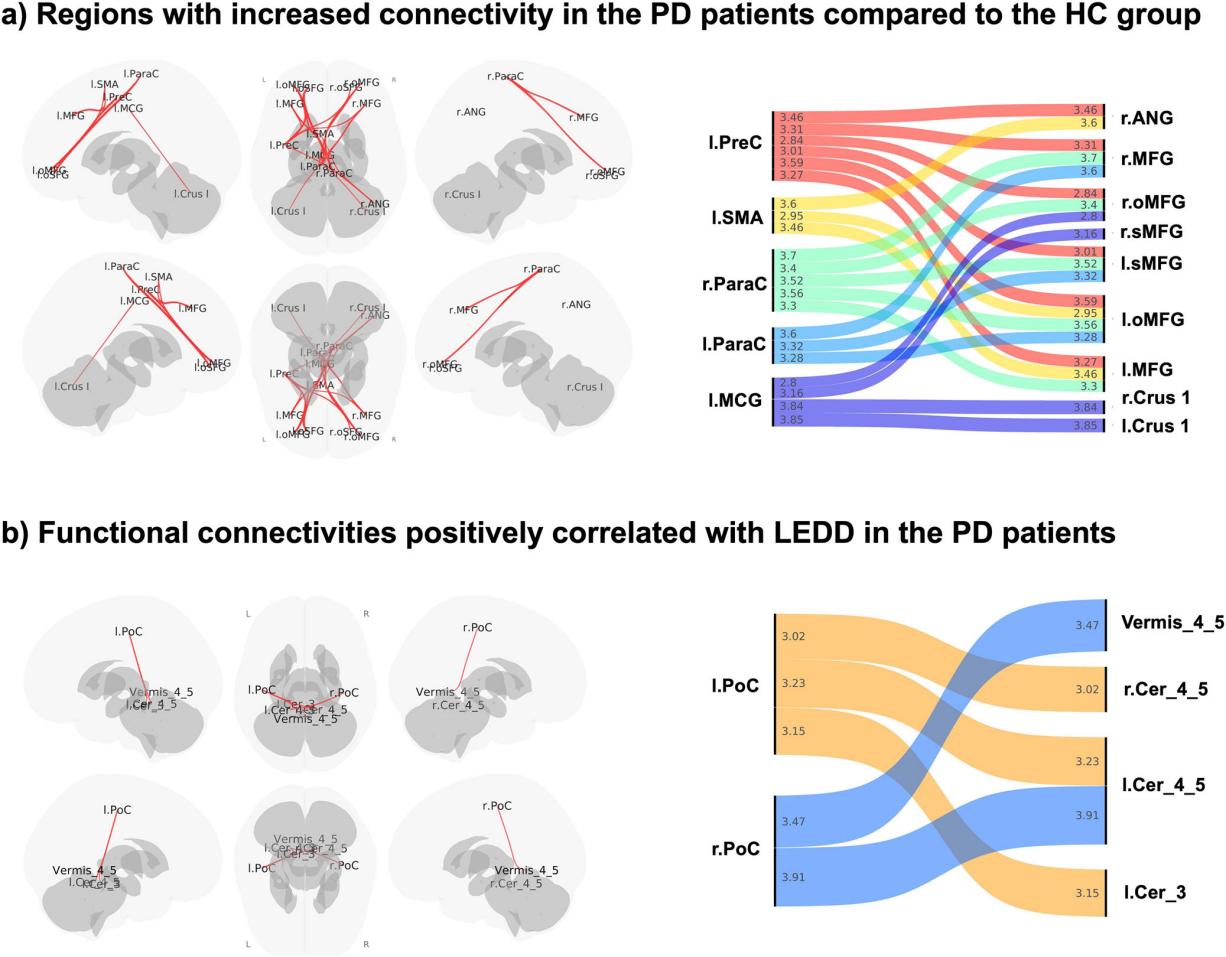
Connectivity alterations in Parkinson's disease (PD). Significant connectivity changes between the PD and healthy control (HC) groups (**a**). In PD patients, further analyses revealed correlations between functional connectivity and Levodopa Equivalent Daily Dose (LEDD) (**b**). In the right panels of both figures, the direction of inter-regional connectivity is displayed, along with the t-values corresponding to these connections, indicated by the numbers within the lines. All connectivities were significantly higher in the PD group compared to the HC group. ANG: Angular gyrus, Cer_3: lobule III of cerebellar hemisphere, Cer_4_5: lobule IV and V of cerebellar hemisphere, Crus I: Crus I of cerebellar hemisphere, MCG: Middle cingulate gyrus, MFG: Middle frontal gyrus, mSFG: Superior frontal gyrus, medial part, oMFG: Middle frontal gyrus, orbital part, oSFG: Superior frontal gyrus, orbital part, ParaCG: Paracentral gyrus, PoCG: Postcentral gyrus, PreCG: Precentral gyrus, SFG: Superior frontal gyrus, SMA: Supplementary motor area, Vermis 4_5: Lobule IV and V of vermiş. L: Left, R: Right.

In the whole-brain ROI-to-ROI comparison between the HC and PD groups, two clusters with significant connectivity changes were identified (Table 2). The first cluster included the right paracentral lobule, bilateral middle frontal gyrus, bilateral orbital middle frontal gyrus, bilateral orbital superior frontal gyrus, left precentral gyrus, right angular gyrus, left middle cingulate gyrus, and left supplementary motor area (F(4,141) = 6.45; p-FDR = 0.008). The second cluster comprised the left middle cingulate gyrus and bilateral cerebellum Crus I (F(4,141) = 5.47; p-FDR = 0.018) (Fig. 1a). All connectivities were significantly higher in the PD group compared to the HC group.

**Table 2:** Increased connectivity in Parkinson’s disease group compared to healthy controls

	T (144)	p-FDR
**Cluster 1**		
R paracentral lobule – R middle frontal gyrus	3.70	0.011
R paracentral lobule – L middle frontal gyrus, orbital	3.56	0.011
R paracentral lobule – L superior frontal gyrus, orbital	3.52	0.011
R paracentral lobule – R middle frontal gyrus, orbital	3.40	0.013
L precentral gyrus – L middle frontal gyrus, orbital	3.59	0.014
L precentral gyrus – R angular gyrus	3.46	0.014
R paracentral lobule – L middle frontal gyrus	3.30	0.014
L precentral gyrus – R middle frontal gyrus	3.31	0.016
L precentral gyrus – L middle frontal gyrus	3.27	0.016
L middle cingulate gyrus – L superior frontal gyrus, orbital	3.16	0.016
L supplementary motor area – R angular gyrus	3.60	0.021
L supplementary motor area – L middle frontal gyrus	3.46	0.021
L paracentral lobule – R middle frontal gyrus	3.60	0.025
L paracentral lobule – R superior frontal gyrus, orbital	3.32	0.025
L paracentral lobule – L middle frontal gyrus, orbital	3.28	0.025
L precentral gyrus – L superior frontal gyrus, orbital	3.01	0.026
L supplementary motor area – L middle frontal gyrus, orbital	2.95	0.031
L middle cingulate gyrus – R middle frontal gyrus, orbital	2.80	0.034
L precentral gyrus – R middle frontal gyrus, orbital	2.84	0.038
**Cluster 2**		
L middle cingulate gyrus – L cerebellum crus I	3.85	0.004
L middle cingulate gyrus – R cerebellum crus I	3.84	0.004

R: right, L: left.

### The functional connectivity of the habenula

**Fig. 2:**
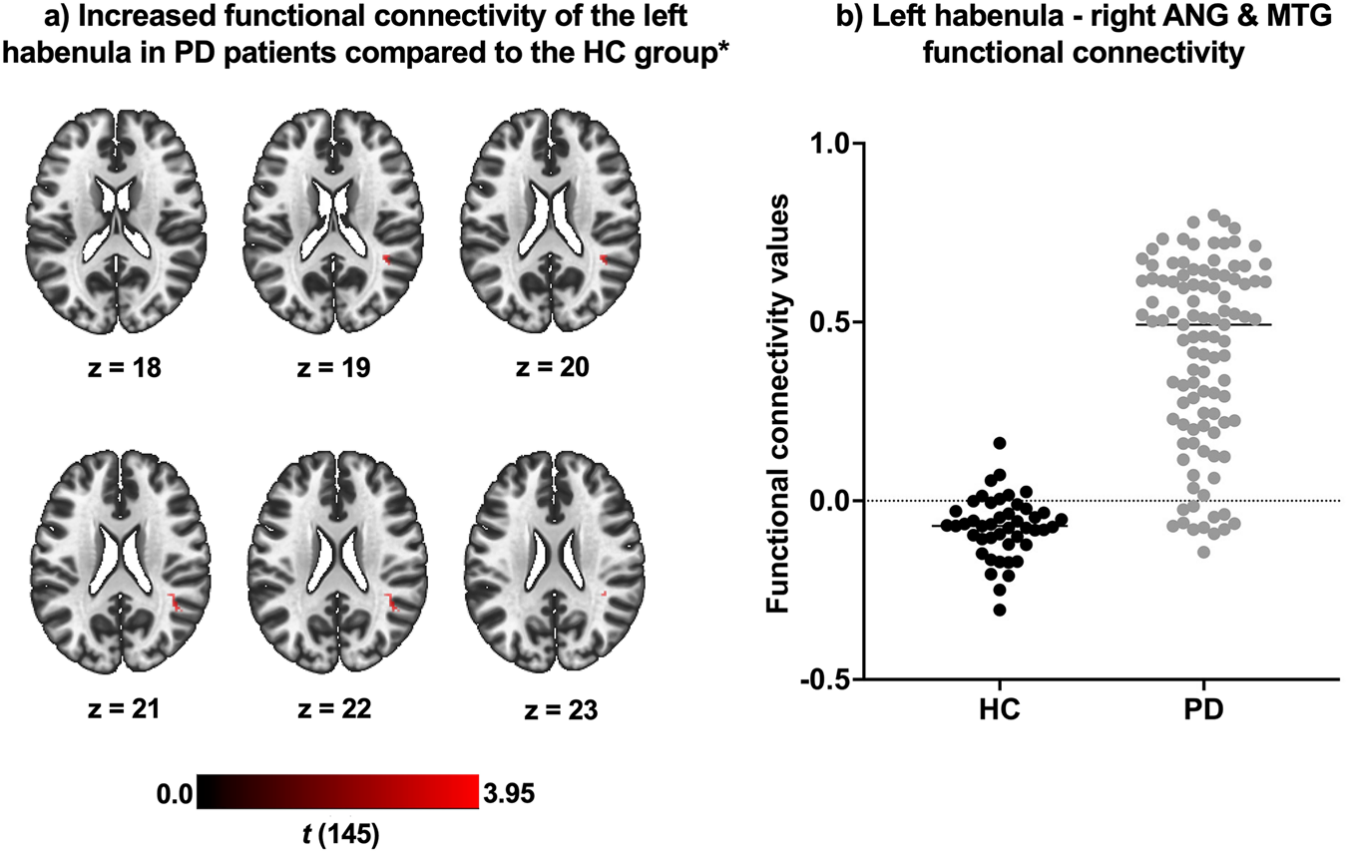
Altered habenula connectivity in Parkinson's disease (PD). **a** The cluster located at the intersection of the right angular gyrus and middle temporal gyrus, which shows increased connectivity with the left habenula in the PD group compared to the healthy controls (HC) group. **b** The functional connectivity values of the cluster, indicating enhanced connectivity with the left habenula in PD patients. The mean functional connectivity value in the HC group is -0.07 (indicating almost no connectivity), while the functional connectivity value in the PD group is 0.41. In summary, while there is nearly no connectivity between the right angular gyrus and the middle temporal gyrus in the HC group, this connectivity was found to be significantly increased in the PD group. *Adjusted for age, antidepressant use and Beck Depression Inventory total scores. ANG: Angular gyrus, MTG: Middle temporal gyrus.

The seed-to-voxel analysis comparing the two groups in terms of Hb connectivity revealed that PD patients exhibited increased functional connectivity of the left Hb compared to the HC group. This increase was observed in a cluster located at the intersection of the right middle temporal gyrus and angular gyrus (MNI coordinates: x = 40, y = -40, z = 22; cluster size = 24 voxels; t(144) = 3.95; p-FDR = 0.011) (Fig. 2). No significant differences were detected between the two groups regarding the functional connectivity of the right Hb.

### Correlations between functional connectivity, BDI scores, and clinical features in PD group

In the whole-brain ROI-to-ROI analysis of the PD group, the analysis of functional connectivity correlating with LEDD revealed a cluster with a positive correlation (F(3, 96) = 7.88; p-FDR = 0.020) (Table 3). This cluster included the bilateral postcentral gyrus, bilateral lobules IV and V of the cerebellar hemisphere, lobules IV and V of the vermis, and left lobule III of the cerebellar hemisphere (Fig. 1b). No functional connectivity was found to be negatively correlated with LEDD. There was no correlation with functional connectivity and the other clinical features as BDI total score, MoCa total score, MDS-UPDRS Part III, and disease duration.

**Table 3:** Functional connectivity positively correlated with levodopa equivalent daily dose in Parkinson’s disease group

	T (98)	p-FDR
**Cluster 1**		
R postcentral – L lobule IV and V of cerebellar hemisphere	3.91	0.010
R postcentral – Lobule IV and V of vermis	3.47	0.022
R postcentral – L lobule III of cerebellar hemisphere	3.15	0.031
L postcentral – L lobule IV and V of cerebellar hemisphere	3.23	0.031
L postcentral – R lobule IV and V of cerebellar hemisphere	3.02	0.038

R: Right, L: Left.

No correlation was found between habenula connectivity and BDI total score, MoCA total score, LEDD, MDS-UPDRS Part III score, or disease duration within the PD group.

## Discussion

This study examined whole-brain and Hb functional connectivity and its links to clinical features in early-stage PD patients compared to HCs. We found altered left Hb connectivity with the right middle temporal and angular gyri, as well as functional changes in brain areas linked to motor and cognitive functions. Connectivity alterations in postcentral gyri and cerebellum correlated with LEDD, reflecting early neurobiological changes in PD. These findings suggest widespread neural network disruptions beyond the nigrostriatal pathway, potentially contributing to early motor and cognitive symptoms in PD.

Depression, including mild forms like SubD, is a common non-motor symptom in PD, affecting 8% to 28.8% of patients and posing diagnostic challenges due to its overlap with PD’s motor symptoms^26^. In the present study, PD patients demonstrated higher BDI scores than HCs. There were only 5 PD patients who used antidepressant or anxiolytic treatments. However, SubD was identified in 32.7% of PD patients compared to 11.1% in the HC group. Higher BDI scores were significantly associated with increased LEDD, longer disease duration, and lower MoCA scores, but showed no correlation with H&Y stages or MDS-UPDRS Part III scores. These findings highlight the early prevalence of depression in PD and its association with disease progression and cognitive decline.

The whole-brain analysis identified two clusters with altered connectivity in PD patients. One cluster showed increased connectivity involving motor and cognitive regions such as the paracentral lobule, precentral gyrus, middle cingulate gyrus, supplementary motor area (SMA), superior and middle frontal gyri, and angular gyrus. These areas are integral to motor control, movement planning, postural stability, and cognitive-emotional functions^27^. Dysfunction in these regions is linked to common PD symptoms, including gait and balance difficulties, impaired voluntary movement initiation, apathy, depression, and decision-making challenges. Tuovinen et al.^10^ investigated longitudinal functional connectivity changes in PD and found increased connectivity between the cerebellum and the somatomotor network, along with decreased connectivity between motor regions such as the Rolandic operculum, precentral gyrus, SMA, postcentral gyrus, and middle cingulate cortex in early disease stages. Other studies have also reported both decreased and increased functional connectivity within these areas in PD compared to HCs^27,28^. These regions are part of a broader motor network that includes the basal ganglia, thalamus, and various cortical areas. PD primarily affects the basal ganglia, disrupting dopamine signaling and altering motor circuit outputs to cortical areas like the SMA and precentral gyrus. This disruption not only results in the characteristic motor symptoms but also influences cognitive and emotional aspects of movement. While decreased connectivity often reflects neural dysfunction, increased connectivity may indicate compensatory mechanisms. In our study, increased connectivity between motor and cognitive regions suggests the brain’s plasticity in adapting to neurodegeneration by enhancing integration across networks to maintain function.

The second cluster showed increased connectivity between the middle cingulate gyrus and cerebellum Crus I. The middle cingulate gyrus is involved in motor control, pain perception, decision-making, and cognitive processes, while the cerebellum contributes to motor execution, learning, gait, and non-motor functions like cognition^29,30^. Crus I, specifically, is linked to executive functions such as working memory, planning, visuomotor integration, and refining voluntary movements^30–33^. Increased connectivity with Crus I may reflect its role in integrating motor and cognitive processes, adapting to neurodegeneration in PD. Similar findings in prior studies revealed altered cerebellar connectivity with motor and cognitive networks, with increased sensorimotor cerebellar connectivity associated with worsening motor outcomes over time^9,10^. Previous studies have also noted increased cerebellar connectivity in PD patients compared to controls, as well as disrupted communication between the cerebellum and other areas of the sensorimotor network^34,35^. Our results suggest that the enhanced connectivity between the middle cingulate gyrus and Crus I represents a compensatory mechanism, supporting the cerebellum’s expanded role in addressing the motor and cognitive challenges of PD. This adaptation highlights the cerebellum’s importance in maintaining complex motor and cognitive functions amidst PD-related disruptions.

Functional neuroimaging studies are crucial for elucidating the contributions of structures beyond the basal ganglia to the pathophysiology of PD. To date, only one study has examined Hb connectivity in PD, finding increased connectivity between the Hb and the amygdala, thalamus, and striatum bilaterally, along with decreased connectivity between the bilateral Hb and the left frontal and precentral cortices in PD patients with punding compared to those without and HCs^36^. Our study revealed increased connectivity between the left Hb and the right middle temporal and angular gyri in PD patients. These regions, integral to language comprehension, socio-emotional processing, spatial awareness, and multimodal integration, are key components of the default mode network (DMN), which is often disrupted in PD and other disorders^37–39^. Disrupted DMN activity in PD, linked to dopamine deficiency, may underlie executive dysfunction^40^. Animal studies also suggest Hb-related DMN disruptions impact connectivity and cognition^7,41^. Our findings of increased Hb connectivity with these regions likely reflect compensatory mechanisms or early maladaptive network reorganization in response to dopaminergic deficits, potentially serving as markers of subtle cognitive changes in early-stage PD.

The relationship between functional connectivity changes and clinical features in PD remains complex. While our study did not find significant correlations between Hb connectivity and features like BDI scores, MoCA scores, MDS-UPDRS Part III scores, or disease duration, whole-brain analysis revealed notable correlations with LEDD. Prior research has linked Hb connectivity changes to SubD in regions such as the amygdala, insula, PFC, mid-cingulate cortex, and entorhinal cortex^42^. We also did not observe a relationship between whole brain connectivity and BDI scores. There are many studies showing reduced connectivity in the medial frontal gyrus, paracentral lobule, precentral gyrus, and SMA in depressed PD patients, which are associated with motor and emotional impairments^43–47^. The precentral gyrus, crucial for initiating voluntary movement, is further burdened in PD by dopaminergic deficits. As depression severity increases, diminished connectivity between motor and frontal cognitive regions may exacerbate motor symptoms like bradykinesia and impair executive functions. These disruptions highlight the intertwined nature of motor and depressive symptoms in PD and underscore the importance of addressing depression as part of PD management^48^. Contrary to our hypothesis, altered habenular connectivity were not significantly correlated with BDI or MoCA scores. This finding suggests that Hb connectivity alterations may reflect mechanisms distinct from depression and cognitive impairment. One possible explanation is that Hb dysfunction in PD may be related to broader motivational, autonomic, or reward-processing abnormalities, which are not fully captured by standard depression or cognitive screening tools. BDI, while widely used in PD research, is a self-report measure that provides only a brief snapshot of depressive symptoms and may not fully capture the complexity of mood disturbances in PD. The BDI does not differentiate between somatic symptoms related to PD (e.g., fatigue, motor slowing) and primary affective symptoms, which could influence the interpretation of our findings. Additionally, we observed that significant connectivity alterations were lateralized to the left Hb, aligning with previous research suggesting potential hemispheric asymmetries in Hb function. While lateralization of habenular activity remains incompletely understood, some studies indicate that the left Hb may be preferentially involved in cognitive and emotional regulation, while the right Hb has stronger links to stress-related responses^23,49^. This asymmetry could suggest that early-stage PD-related connectivity changes preferentially affect cognitive-emotional circuits rather than stress-related pathways. Instead of concluding that Hb connectivity is independent of depression in PD, we acknowledge that multiple factors may have contributed to this negative result, including sample characteristics (mild depressive symptoms in our cohort), potential nonlinear relationships, and interindividual variability in disease progression. This highlights the need for larger, longitudinal studies with detailed neuropsychiatric assessments to better understand the role of Hb connectivity in mood and cognitive symptoms in PD. Additionally, while rs-fMRI provides valuable insights into intrinsic connectivity patterns, it does not capture dynamic task-dependent activity. Given the Hb’s role in reward processing, aversion, and decision-making, future studies utilizing task-based fMRI paradigms—particularly those probing reward anticipation, effort-based decision-making, and emotional regulation—may offer greater specificity in understanding Hb function in PD.

While Hb connectivity showed no significant correlation with LEDD, our whole-brain analysis revealed a positive correlation between LEDD and increased connectivity of the bilateral postcentral gyrus with cerebellar lobules III-IV-V and the vermis (lobules IV-V). The postcentral gyrus, as the primary somatosensory cortex, processes sensory inputs and integrates them with motor commands, while the cerebellum refines motor movements through sensorimotor processing^50^. The functional relationship between the cerebellum and the postcentral gyrus facilitates the integration of sensory data for smooth, coordinated movement. Increased cortico-cerebellar connectivity in PD has been linked to compensatory responses to basal ganglia dysfunction, particularly in patients with postural instability-gait difficulty^29,51,52^. Studies, including the earliest study by Wu et al.^53^ on the effects of L-DOPA, have shown that L-DOPA partially normalizes cerebellar overactivity, which arises as a compensatory response to basal ganglia abnormalities. Esposito et al.^28^ further examined this by conducting a placebo-controlled study with drug-naïve PD patients, and similarly demonstrated that L-DOPA enhances basal ganglia and thalamic involvement in sensorimotor networks. In our study, higher LEDD was associated with increased connectivity between the postcentral gyrus and motor cerebellum, suggesting dopaminergic medication enhances sensory-motor integration. While this connectivity boost may aid motor function, it might also lead to overactivity or maladaptive plasticity, contributing to motor complications like dyskinesias with prolonged treatment. These findings highlight the dual nature of dopaminergic therapy in PD: while improving motor symptoms, it may also reshape brain networks in ways that require careful management. Future research should explore the long-term effects of these connectivity changes and develop strategies to balance motor symptom relief with the minimization of adverse effects. Also, since our PD cohort was scanned while on medication, these findings highlight the importance of considering treatment effects when interpreting connectivity changes. Future studies incorporating OFF-state imaging could help disentangle disease-related and medication-induced alterations in functional connectivity.

The cross-sectional design of this study limits our ability to draw causal conclusions regarding the observed connectivity changes. Without longitudinal data, it remains unclear how these alterations evolve and influence clinical outcomes over time. Future research should focus on longitudinal studies to track these changes and their impact on disease progression. Additionally, investigating the functional significance of Hb connectivity changes in relation to specific cognitive and emotional processes in PD will be crucial for understanding their role in the broader context of disease pathology and symptomatology. While the current study employed a seed-based connectivity approach via the GLM to test specific hypotheses regarding habenular connectivity in early-stage PD, future studies could benefit from the complementary use of Independent Component Analysis for exploratory network analysis. Our study has also another limitation related to the male-to-female ratio and the lack of detailed ancestry data. Males are approximately 1.5 times more likely to develop PD than females, but emerging evidence suggests that sex differences influence functional connectivity and non-motor symptoms, with women showing higher anxiety and autonomic dysfunction, and men exhibiting greater cognitive impairment^54–58^. Regarding ancestry demographics, our study predominantly included participants of European descent, which may limit generalizability across populations with different genetic and environmental risk factors for PD^59,60^. Future studies with more balanced sex ratios and diverse populations are needed to explore these differences and improve the generalizability of our findings. Despite these limitations, a notable strength of this study is that it marks the first comprehensive exploration of Hb functional connectivity in PD patients, leveraging the capabilities of advanced 7 T MRI technology. The use of 7 T MRI was crucial in detecting the laterality of habenular connectivity changes, which may reflect adaptive network reorganization or compensatory mechanisms in early PD. Its high spatial resolution and enhanced contrast allowed for a more precise mapping of habenular connectivity with both cortical and subcortical regions, revealing subtle network alterations that may not be as easily detected with lower field strengths^25^. These findings underscore the importance of ultra-high-field imaging not only in advancing our understanding of habenular function in PD but also in developing imaging biomarkers for early diagnosis, disease tracking, and therapeutic response assessment.

Our findings provide novel insights into the functional connectivity alterations of the Hb and cortical regions in early-stage PD. This study is the first to investigate Hb connectivity in a large cohort of early PD patients. We observed increased connectivity between the left Hb and the right middle temporal and angular gyri, alongside widespread cortical connectivity changes, highlighting the complex neural alterations occurring early in PD. This increased connectivity between the Hb and cognition-related regions may serve as early markers for cognitive decline in PD. Furthermore, the altered connectivity between motor regions and the cerebellum underscores the efforts to compensate for dopamine loss, reflecting both the benefits and risks associated with dopaminergic therapy. Recognizing these connectivity changes is crucial for optimizing treatment and understanding PD progression. These findings emphasize that PD extends beyond basal ganglia dysfunction to involve extensive network disruptions. Future longitudinal research is needed to further explore these connectivity changes and refine therapeutic strategies to alleviate symptoms and enhance the quality of life for individuals with PD.

## Methods

### Participant selection

The study complied with the Declaration of Helsinki and the Medical Research Involving Human Subjects Act and received ethical approval from the Institutional Review Board (IRB) of Maastricht University Medical Centre (METC AZM/UM 18-027). Written informed consent was obtained from all participants before inclusion.

TRACK-PD is a longitudinal observational study using 7 T MRI in newly diagnosed PD patients^24^. The study aims to provide valuable insights into the diverse clinical presentations of PD and their associated MRI features. Its primary objective is to improve the understanding of PD and identify novel biomarkers for tracking disease progression, ultimately facilitating the development of new therapeutic strategies. The inclusion criteria for the TRACK-PD study were as follows: (a) A Parkinson’s disease diagnosis confirmed by a neurologist within the past 3 years following established criteria^61^, (b) A baseline Montreal Cognitive Assessment (MoCA) score of ≥24 with no evidence of dementia, (c) Proficiency in Dutch, (d) Age 18 or older and (e) Written informed consent to participate. Participants with contraindications for a 7 T MRI scan were excluded. All PD participants underwent MRI while on their usual dopaminergic medication regimen (on state). This ensures that observed functional connectivity changes reflect disease-related and medication-related effects, rather than acute withdrawal from dopaminergic therapy.

The TRACK-PD study included 106 PD patients and 45 HCs. Following MRI quality control, 104 PD patients (66 males, 18 females) and 45 HCs (31 males, 14 females) were retained. Clinical and demographic data were collected, including age, sex, handedness, disease duration, Hoehn & Yahr stage (H&Y)^62^, Movement Disorders Society-Unified Parkinson’s Disease Rating Scale (MDS-UPDRS) Part III (medication-on)^63^, total levodopa equivalent daily dose (LEDD)^64^, and MoCA scores^65^.

Depression levels were evaluated using the Beck Depression Inventory (BDI), a 21-item questionnaire^66^. The BDI assessment and MRI scanning were conducted at the baseline visit to ensure temporal consistency between clinical and neuroimaging data. The cut-off values applied were specifically tailored for PD, consistent with previous studies that also utilized BDI. Scores were categorized as follows: 0-8 indicated no depression, 9-15 represented subthreshold depression (SubD), and ≥16 signified significant depression^26,67^.

### MRI acquisition and preprocessing

Participants underwent scanning with a 7 T MRI scanner (Magnetom, Siemens, Erlangen, Germany) using a Nova Medical 32-channel head coil, enhanced by dielectric pads to improve signal in temporal brain regions^68^.

The whole-brain resting-state fMRI scan consisted of 280 volumes, with an additional five volumes recorded using a reverse phase-encoding direction. During the scan, participants were instructed to focus on a crosshair projected on a screen and to let their minds wander without concentrating on any specific thoughts. The fMRI scan parameters were as follows: echo time (TE) of 18.6 ms, repetition time (TR) of 2000 ms, flip angle of 70°, field of view (FoV) of 200 × 200 mm, resolution of 1.25 × 1.25 × 1.25 mm³, 92 slices with an axial orientation, and a total acquisition time of 11:12 minutes^24^.

The results presented in this manuscript were derived using CONN release 22.a^69^ and SPM release 12.7771^70^. The functional and anatomical data underwent pre-processing through a flexible pipeline that involved several steps: realignment with correction for susceptibility-distortion interactions, outlier detection, indirect segmentation and MNI space normalization, and smoothing. Each scan was co-registered to a reference image (the first scan of the first session) using a least-squares approach with a 6-parameter (rigid body) transformation and resampled with B-spline interpolation to correct for motion and magnetization interactions. Potential outlier scans were identified using ART as scans with frame shifts greater than 0.9 mm or global BOLD signal changes greater than 5 standard deviations, and a reference BOLD image was calculated for each subject by averaging all scans without outliers. Finally, the functional data were spatially smoothed using a 2.5 mm full-width half-maximum (FWHM) Gaussian kernel.

Functional data were further denoised using a standard pipeline^71^. This process included regression of potential confounding effects characterized by white matter time series, cerebrospinal fluid (CSF) time series, motion parameters and their first-order derivatives, outlier scans, session effects and their first-order derivatives, and linear trends within each functional run, followed by band-pass frequency filtering of the BOLD time series between 0.008 Hz and 0.09 Hz. Within each subject’s eroded segmentation mask, CompCor^72,73^ noise components within white matter and CSF were estimated by calculating the mean BOLD signal and the largest principal components orthogonal to the mean BOLD, motion parameters and outlier scans.

### Habenula and whole brain functional connectivity

For the whole-brain functional connectivity analysis, ROI-ROI connectivity matrices were estimated to characterize the functional connectivity between each pair of regions among 118 ROIs. This set of ROIs included 116 brain regions from the AAL atlas^74^ and the right and left Hb as additional regions. The AAL atlas was selected because it provides a comprehensive and widely used parcellation of the brain into 116 regions.

For the analysis of habenular functional connectivity, seed-based connectivity maps were created to delineate the spatial patterns of functional connectivity with the seed regions. The seed regions, which were manually segmented for each participant, comprised the right and left Hb, as previously detailed^75^. The association between the BOLD signal time series of each seed area and the target voxels was modelled by a Fisher-transformed bivariate correlation coefficient from a weighted general linear model estimated separately for each seed area and the target voxels.

### Statistical analysis

Statistical analyses were performed using SPSS 28.0 (IBM, SPSS Inc., New York, United States). Normality was confirmed using the Kolmogorov-Smirnov method. An independent sample t-test or one-way analysis of variance (ANOVA) was used for normally distributed data, whereas the Kruskal-Wallis H-test or Mann-Whitney U-test was used for non-normally distributed variables for group comparisons.

Group-level analyses of habenular functional connectivity were conducted using a general linear model (GLM). For each voxel, a separate GLM was estimated with the first-level connectivity measures at that voxel as the dependent variable and the group as the independent variable. Age, BDI scores and antidepressant use were included as covariates when comparing HC and PD groups. Multivariate parametric statistics with random effects across subjects and sample covariance estimation across multiple measures were employed to evaluate hypotheses at the voxel level. Inferences were drawn at the level of individual clusters of interest. Results were thresholded using a connection significance of *p* < 0.001, combined with a cluster threshold of p-FDR < 0.05 for multiple comparison.

To assess whole brain functional connectivity, the study used a multivariate pattern analysis (MVPA) omnibus test with the CONN toolbox. This approach identifies significant patterns of connectivity across many regions of interest (ROIs), ensuring that both individual connections and clusters of connections match the established significance criteria. In comparing HC and PD groups, age, BDI scores and antidepressant use were included as covariates. We selected age and antidepressant use as covariates based on their potential influence on both functional connectivity and clinical measures. Age was included to account for its known effects on brain connectivity and cognitive performance^76^. We included antidepressant use as a covariate due to its potential effects on mood-related brain networks, ensuring that any variability introduced by its use was accounted for^77^. A statistical criterion of *p* < 0.05 was chosen for connection-level inferences, whereas a more stringent cluster-level correction of p-FDR < 0.05 was utilized to account for multiple comparisons^78^.

To compare the functional connectivity of the Hb and whole brain between HC and PD groups, and to identify connectivity correlated with BDI, LEDD, UPDRS-III scores specifically in the PD group, a MVPA omnibus test was performed as previously described. Covariates were chosen based on the specific correlation being examined: age, LEDD, antidepressant use, and UPDRS-III scores were used as covariates for BDI scores; age, BDI, antidepressant use, and UPDRS-III scores were used as covariates for LEDD scores; age, BDI, antidepressant use and LEDD scores were used as covariates for UPDRS-III scores. This approach ensured that relevant confounders were controlled for correlation analyses.

## Data Availability

The data supporting the findings of this study are available on request from the corresponding author. The data are not publicly available due to privacy or ethical restrictions.
